# HPTN 083‐02: factors influencing adherence to injectable PrEP and retention in an injectable PrEP study

**DOI:** 10.1002/jia2.26252

**Published:** 2024-05-23

**Authors:** Christina Psaros, Georgia R. Goodman, Jasper S. Lee, Whitney Rice, Colleen F. Kelley, Temitope Oyedele, Lara E. Coelho, Nittaya Phanuphak, Yashna Singh, Keren Middelkoop, Sam Griffith, Marybeth McCauley, James Rooney, Alex R. Rinehart, Jesse Clark, Vivian Go, Jeremy Sugarman, Sheldon D. Fields, Adeola Adeyeye, Beatriz Grinsztejn, Raphael J. Landovitz, Steven A. Safren, the HPTN 083‐02 Study Team

**Affiliations:** ^1^ Department of Psychiatry Massachusetts General Hospital Boston Massachusetts USA; ^2^ Harvard Medical School Boston Massachusetts USA; ^3^ Department of Emergency Medicine Brigham and Women's Hospital Boston Massachusetts USA; ^4^ Department of Behavioral Social and Health Education Sciences Emory University Rollins School of Public Health Atlanta Georgia USA; ^5^ Division of Infectious Diseases Department of Medicine Emory University School of Medicine Atlanta Georgia USA; ^6^ AstraZeneca Pharmaceuticals Chicago Illinois USA; ^7^ Instituto Nacional de Infectologia Evandro Chagas‐Fiocruz Rio de Janeiro Brazil; ^8^ Institute of HIV Research and Innovation Bangkok Thailand; ^9^ Center of Excellence in Transgender Health Chulalongkorn University Bangkok Thailand; ^10^ Desmond Tutu HIV Centre University of Cape Town Cape Town South Africa; ^11^ Department of Medicine University of Cape Town Cape Town South Africa; ^12^ Family Health International 360 Washington DC USA; ^13^ Gilead Sciences Foster City California USA; ^14^ ViiV Healthcare Durham North Carolina USA; ^15^ Division of Infectious Diseases Department of Medicine David Geffen School of Medicine University of California Los Angeles California USA; ^16^ Department of Health Behavior University of North Carolina Gillings School of Global Public Health Chapel Hill North Carolina USA; ^17^ Berman Institute of Bioethics and School of Medicine Johns Hopkins University Baltimore Maryland USA; ^18^ Ross and Carol Nese College of Nursing The Pennsylvania State University University Park Pennsylvania USA; ^19^ Nigeria Country Office US Centers for Disease Control and Prevention Abuja Nigeria; ^20^ Center for Clinical AIDS Research and Education David Geffen School of Medicine University of California Los Angeles California USA; ^21^ Department of Psychology University of Miami Miami Florida USA

**Keywords:** HIV prevention, injectable PrEP, men who have sex with men, pre‐exposure prophylaxis, qualitative, transgender women

## Abstract

**Introduction:**

HPTN 083 demonstrated the superiority of long‐acting cabotegravir (CAB‐LA) versus daily oral emtricitabine/tenofovir disoproxil fumarate (TDF/FTC) as pre‐exposure prophylaxis (PrEP) among cisgender men and transgender women who have sex with men (MSM/TGW). HPTN 083 provided the first opportunity to understand experiences with injectable PrEP in a clinical trial.

**Methods:**

Participants from two US sites (Chicago, IL and Atlanta, GA) and one international site (Rio de Janeiro, Brazil) were purposively sampled for individual qualitative interviews (*N* = 40), between November 2019 and March 2020, to explore trial experiences, barriers to adherence and other factors that may have impacted study implementation or outcomes. The blinded phase ended early due to efficacy; this analysis includes interviews conducted prior to unblinding with three groups defined by adherence (i.e. injection visit attendance): adherent (*n* = 27), non‐adherent (*n =* 12) and early discontinuers (*n* = 1). Data were organized using NVivo software and analysed using content analysis.

**Results:**

Participants (mean age: 27) were primarily cisgender MSM (90%) and Black/African American (60%). Reasons for trial enrolment and PrEP use included a preference for using HIV prevention medication versus treatment in the event of HIV acquisition; the ability to enhance health via study‐related education and services; access to a novel, convenient HIV prevention product at no cost; and contributing to MSM/TGW communities through research. Participants contrasted positive experiences with study staff with their routine clinical care, and emphasized increased scheduling flexibility, thorough communication, non‐judgemental counselling and open, affirming environments (e.g. compassion, less stigma) as adherence facilitators. Injection experiences were positive overall; some described early injection‐related anxiety, which abated with time and when given some measure of control (e.g. pre‐injection countdown), and minimal injection site discomfort. Some concerns and misperceptions about injectable PrEP were reported. Barriers to adherence, across all adherence categories, included structural factors (e.g. financial constraints, travel) and competing demands (e.g. work schedules).

**Conclusions:**

Respondents viewed injectable PrEP trial participation as a positive experience and a means of enhancing wellbeing. Study site flexibility and affirming clinic environments, inclusive of non‐judgemental counselling, were key facilitators of adherence. To support injection persistence, interventions that address structural barriers and promote flexible means of injection delivery may be most effective.

## 1 INTRODUCTION

HIV Prevention Trials Network (HPTN) 083 was the first large‐scale, randomized, double‐blind efficacy trial of long‐acting injectable cabotegravir (CAB‐LA) versus daily oral emtricitabine/tenofovir disoproxil fumarate (TDF/FTC) as pre‐exposure prophylaxis (PrEP) for HIV prevention [1]. The trial was conducted among cisgender men and transgender women who have sex with men (MSM/TGW) in the United States, Latin America, Africa and Asia (*N* = 4566). In May 2020, the blinded phase was stopped early due to efficacy, following a Data and Safety Monitoring Board (DSMB) review that determined injectable CAB‐LA demonstrated superiority over TDF/FTC on HIV incidence [1]. Of note, 86% of TDF/FTC arm participants had detectable tenofovir in plasma; therefore, while imperfect, adherence did not interfere with the ability to evaluate product efficacy, as in other HIV prevention trials [2, 3].

Challenges associated with daily oral PrEP adherence encompass individual barriers (e.g. lifestyle factors, substance use, mental health comorbidities), social and interpersonal barriers (e.g. stigma, intimate partner attitudes) and structural barriers (e.g. financial constraints, lack of access or education) [4, 5, 6, 7]. While injectable PrEP may offer effective HIV prevention without certain adherence barriers, data on experiences with PrEP injections are limited. A subset of HPTN 083 participants were enrolled in a qualitative sub‐study (HPTN 083‐02), pre‐unblinding, to explore overall trial experiences, barriers to completing study procedures and whether other behavioural, contextual or individual variables affected trial implementation or outcomes.

## 2 METHODS

### 2.1 Participants and recruitment

#### 2.1.1 Eligibility and classification of participants

Eligibility criteria for the qualitative sub‐study (HPTN 083‐02) mirrored those of the parent HPTN 083 study. In brief, participants were adults (≥18 years old), assigned male at birth and currently identifying as either cisgender men or TGW who have sex with men, HIV negative at enrolment, at high risk for sexually acquiring HIV, and in general good health per clinical and laboratory assessments. Full inclusion and exclusion criteria are reported elsewhere [1]. Sub‐study participants were purposively sampled based on three adherence classifications in order to maximize variation (Table 1).

**Table 1:** Definitions of adherence classifications^a^

Group	Definition
Adherent	Individuals who received at least two consecutive injections within 10 weeks of their prior injection (the timeline of which began once injections were scheduled every 8 weeks), at any point during the injection phase.
Non‐adherent	Individuals who received any injection more than 10 weeks following their prior injection any point during the injection phase, but had not been lost to follow‐up or prematurely left the trial.
Early discontinuer	Individuals who were not actively engaged in the study or were engaged in a way other than described for the adherent and non‐adherent groups (e.g. those who declined additional injections but agreed to additional follow‐up, those lost to follow‐up, those who wished to withdraw from the main trial but agreed to participate in the sub‐study).

^a^ The injection phase trial occurred after a 5‐week oral lead‐in to establish safety and tolerability.

#### 2.1.2 Recruitment

Trained staff reviewed lists of parent study participants and identified eligible individuals to be contacted for the sub‐study. Parent study participants were also referred directly by study clinicians. Interviews were conducted between November 2019 and March 2020. Recruitment was stopped prematurely due to parent study unblinding.

#### 2.1.3 Study sites

Sub‐study participants were recruited from five sites: two US (Chicago, IL and Atlanta, GA) and three international (Rio de Janeiro, Brazil, Cape Town, South Africa and Bangkok, Thailand). Overall anticipated enrolment was *N* = 200−300. Only participants from the three sites that conducted interviews prior to unblinding (Chicago, IL, Atlanta, GA and Rio de Janeiro, Brazil) are included in the present analyses.

### 2.2 Procedures

#### 2.2.1 Approvals and informed consent

The study was approved by the institutional review board and/or ethics committees at each site (Adolescent & Young Adult Research at The CORE Center, Chicago, IL; The Hope Clinic of the Emory Vaccine Center, Decatur, GA; and Instituto de Pesquisa Clinica Evandro Chagas, Rio de Janeiro, Brazil). Written informed consent was obtained from all participants.

#### 2.2.2 Parent study procedures

The overall trial procedures are described by Landovitz et al. [1]. Participants received adherence counselling following the Life‐Steps approach for PrEP [8, 9], a cognitive‐behavioural and motivational interviewing‐based intervention that emphasizes non‐judgementality. All parent study participants received Life‐Steps adherence counselling at baseline and follow‐up visits; individuals who reported adherence concerns received more in‐depth problem‐solving support.

#### 2.2.3 Qualitative interviews

Sub‐study participants completed individual interviews (in‐person or via phone) lasting 30−60 minutes in their native language. Interviewers were trained in qualitative techniques by the sub‐study principal investigators (CP, SS), who also reviewed initial transcripts for quality and training. Interviews were digitally recorded, transcribed, translated into English by local bilingual study staff (as needed), and then reviewed for errors and omissions. Participants were compensated per local standards.

### 2.3 Measures

#### 2.3.1 Socio‐demographics

Socio‐demographic data (i.e. age, race, ethnicity, gender identity, education level, marital status) were abstracted from the parent study database.

#### 2.3.2 Qualitative interview guide

Interview guides were developed in accordance with guidelines outlined by Huberman and Miles [10] and Strauss and Corbin [11]. Interviews explored the following key domains: (1) overall study experiences; (2) experiences with injectable PrEP; (3) facilitators of injection visit attendance; (4) barriers to injection visit attendance; (5) facilitators of pill adherence; (6) barriers to pill adherence; (7) perceptions of provided adherence support; and (8) reasons for study dropout or discontinuation, as applicable. Qualitative data across the first four domains are presented here. Sample questions and probes are provided in Table 2.

**Table 2:** Sample interview domains, questions and probes

Domains	Sample questions and probes
Overall study experiences	How would you describe your overall experience attending study visits? What worked well for you? What didn't work well for you?
Experiences with injectable PrEP	How do you feel about the study injections? Tell me about any pain you have experienced as a result of the injections. How have you managed that? Would you say these visits are easy or difficult? Why?
Facilitators of injection visit attendance	What makes you want to come to your study injection visits? What do you do to avoid forgetting your study injection visits?
Barriers to injection visit attendance	What gets in the way of getting to the clinic for your injection visits? How do you feel about receiving injections for an illness that you do not have? Sometimes peoples’ HIV risk changes over time (e.g. an increase or decrease in sexual activity or partners). How does this affect the way you get to your injection visits? How does the environment at the clinic and study staff affect how you get to your injection visits?

### 2.4 Analyses

#### 2.4.1 Descriptive data

Descriptive statistics on socio‐demographics were calculated to characterize the sub‐study sample.

#### 2.4.2 Qualitative analyses

Interview data were analysed using content analysis, an iterative, multi‐step process as described by Huberman and Miles [10] and Strauss and Corbin [11]. Two trained study team members (CP and GRG) independently reviewed transcripts to generate an overarching framework for data interpretation. The framework included structural codes based on domains and questions from the interview guide, as well as thematic codes generated from interviews. NVivo software (version 12) [12] was used to organize data and facilitate analyses by four independent coders (CP, GRG, AB and JL); 25% of transcripts were double‐coded. The coders iteratively reviewed and compared coding for consistency and resolved discrepancies; an audit trail of coding and thematic frameworks was maintained. Data were then re‐examined, salient content areas were highlighted and extracted, and key findings were reviewed by the study team. An examination of differences across adherence categories and gender identities was attempted; however, no salient differences were detected, possibly because of small numbers of gender minority participants, participants from Brazil and participants with suboptimal adherence. As such, qualitative findings are reported collectively across groups.

## 3 RESULTS

### 3.1 Characteristics of study participants

Forty participants were enrolled in Chicago, Illinois, US (*n* = 19, 47.5%), Atlanta, Georgia, US (*n* = 17, 42.5%) and Rio de Janeiro, Brazil (*n* = 4, 10.0%). The blinded phase of the parent study ended before sub‐study recruitment goals across adherence categories were met, hence most participants were adherent (*n* = 27, 67.5%), some were non‐adherent (*n* = 12, 30.0%) and only one was an early discontinuer (2.5%). Socio‐demographic data are presented in Table 3.

**Table 3:** Socio‐demographic characteristics and descriptive statistics (*N* = 40)

	*n*	%
Age (in years)		
Mean (SD)	27.3 (5.0)	–
Range	19–39	–
Location		
Chicago, IL, United States	19	47.5
Atlanta, GA, United States	17	42.5
Rio de Janeiro, Brazil	4	10.0
Race		
American Indian or Alaska Native	2	5.0
Asian	1	2.5
Black or African American	24	60.0
White	11	27.5
More than one race	2	5.0
Ethnicity		
Hispanic or Latino	8	20.0
Not Hispanic or Latino	32	80.0
Gender identity		
Cisgender man	36	90.0
Transgender woman	2	5.0
Gender queer or variant or non‐conforming	2	5.0
Education		
Primary school	1	2.5
Secondary school	4	10.0
Some college/university or higher	19	47.5
College/university or higher	16	40.0
Marital status		
Married or civil union or legal partnership	1	2.5
Living with primary or main partner	3	7.5
Have primary or main partner, not living together	1	2.5
Single or divorced or widowed	35	87.5
Parent study adherence category		
Adherent	27	67.5
Non‐adherent	12	30.0
Early discontinuer	1	2.5

### 3.2 Qualitative findings

**Figure 1:**
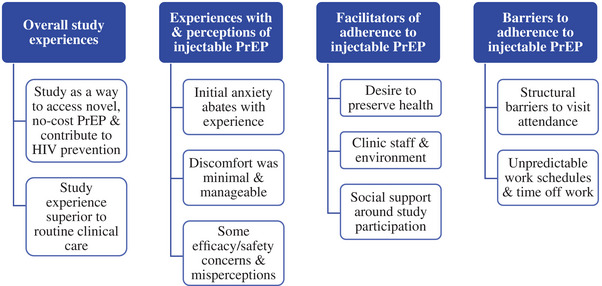
Qualitative domains. *Note: Blue boxes represent overarching domains examined in the qualitative interview guide. White boxes represent emergent subthemes within each domain*.

Qualitative findings were identified across the following domains in the interview guide: (1) overall study experiences; (2) experiences with and perceptions of injectable PrEP; (3) facilitators of adherence to injectable PrEP; and (4) barriers to adherence to injectable PrEP. Emergent subthemes are reported within each domain. Geographic location is specified as “domestic” (i.e. US sites) or “international” in the quotations; country‐level identifiers are not provided to protect participants’ confidentiality. Overall findings are presented in Figure 1.

#### 3.2.1 Overall study experiences: study enrolment was a means to access a novel, convenient HIV prevention tool at no cost and contribute to HIV prevention efforts

Study participation was described as a way to access an innovative HIV prevention product—injectable PrEP—which participants viewed as having the potential to reduce the daily pill‐taking burden of oral PrEP.

*I think [injectable PrEP] is definitely the future of PrEP. You have to go to the hospital or your clinic anyway to get a refill for Truvada, so you might as well just go and get an injectable. Then there's no pill burden*. – Adherent, MSM, Domestic

Participants emphasized that access to both oral and injectable PrEP at no cost was a significant benefit of trial enrolment. Remuneration for participation was also a benefit, particularly for participants who were unemployed and/or facing other financial challenges.

*I was unemployed at the time, and it said like something about getting paid and also starting PrEP, something I had been meaning to do*. – Non‐adherent, MSM, Domestic

Study participation was viewed as a way to enhance overall health and wellbeing due to extra monitoring from medical providers and study staff. Participants expressed that regular testing conducted during study visits—for example blood work to monitor safety in the study—enabled them to more efficiently track their health beyond HIV prevention.

*I became much more health conscious, and… it was very important to me that you guys did the blood work very frequently to know if there was any other underlying issues that we may have missed had I not been part of this program*. – Non‐adherent, MSM, Domestic

*You start to pay attention to things that could often go unnoticed… you will be monitored all the time regarding your health. You take several exams, you are followed up by doctors, if you need psychological support, you will have [it]*. – Adherent, MSM, International

One participant, who identified as TGW, discussed the ways in which study participation indirectly facilitated access to gender‐affirming medical care (via referrals), thereby fostering progress in her gender transition process:

*The study gave me this opportunity of hormonal therapy… I have been discovering many things regarding my transition… The study has made me think much more about my body than before, how it can affect my life*. – Early discontinuer, TGW, International

Finally, participants reported participating in the trial as a means to contribute to the LGBTQ community and/or to HIV prevention research.

*I'm helping advance gay men's health. It's kind of cool to be part of that*. – Non‐adherent, MSM, Domestic

*There's a whole generation that's gone… That's what keeps me coming. You have a pill that can prevent it, and injections, so it's getting closer to hopefully finding a cure*. – Adherent, MSM, Domestic

#### 3.2.2 Overall study experiences: research experience can be superior to routine clinical care

Participants contrasted their study experiences with clinical care—particularly the open, affirming environment at research sites, frequent and thorough communication with study staff and increased flexibility around scheduling study visits versus clinical appointments. Experiences with adherence counselling were positive, with participants describing sessions as non‐judgemental, informative and enlightening. Feedback related to study staff was universally positive, and participants’ relationships with study staff members were key.

*I would like to say these people are my friends… it completes the experience of knowing that I'm helping my community, getting paid, and these people are here for me. They are here to make this study happen, to make health happen*. – Adherent, MSM, Domestic

*It's a relaxed environment… usually the stereotype with hospitals is they're scary, they make you feel like you are a science experiment. I've had some bad experiences at clinics*. – Non‐adherent, MSM, Domestic

Participants described study staff as compassionate and encouraging; staff did not stigmatize them or make value judgements based on sexual behaviour or PrEP use—including during periods of non‐adherence. For many, this was markedly different from other healthcare experiences.

*They made me feel real open… it was a safe space… And it doesn't seem like anyone is judging me. That's the biggest thing. It's kind of a judge‐free, worry‐free zone*. – Adherent, MSM, Domestic

*Being able to talk about sexual encounters… I've learned I don't have to be embarrassed because the staff makes you feel comfortable… It's nice to interact with people that understand the things that you're going through because they're going through similar things*. – Adherent, MSM, Domestic

One participant highlighted a perceived difference between conversations about initiating PrEP in clinical versus research contexts as follows:

*“Hey. I'm interested in getting on PrEP.” A doctor's first question is, “Why?” With the studies, that's not it*. – Adherent, MSM, Domestic

Ease of communication with study staff was also described as a positive part of the study experience, and distinct from participants’ experiences communicating with their non‐study clinical care providers. Participants reported that it was easy to contact study staff with concerns and questions between visits, and that staff provided comprehensive information at each stage. These efforts were enhanced by the staff's attempts to develop strong relationships with participants.

*The outreach is genuine… It feels like, “Hey, I care about you. I care about like your participation in this study, and I'm going to reach out and go that extra step,” like with the text messages or being flexible with appointments*. – Adherent, MSM, Domestic

#### 3.2.3 Experiences with and perceptions of injectable PrEP: initial anxiety abates with experience

Several participants reported injection‐related anxiety early on, including fear of needles, fear of the unknown (i.e. CAB‐LA was identified as a new medication with a new route of delivery) and concerns about the injection site. The desire for injectable PrEP outweighed these fears, and anxiety seemed to dissipate over time and with experience.

*I cannot look at a needle going in my skin. It reminds me of drugs and addicts and things like that, which I have never been, but it's a fear*. – Non‐adherent, MSM, Domestic

*It feels very routine for them and it feels very routine for me as a result of that. I like that kind of normalization*. – Non‐adherent, MSM, Domestic

For one participant, receiving gluteal injections triggered memories of past sexual trauma. However, after two injections, he was able to overcome this reaction and receive injections without issue.

*The first two times I got the shot, it took me back to when I got raped. It was almost just like, okay, I'm bent over. The only difference is that I'm an adult. I can fight for myself now versus being younger, but that's the experience that it jogged. Now, it became a little more normal, so I don't feel that way*. – Non‐adherent, MSM, Domestic

#### 3.2.4 Experiences with and perceptions of injectable PrEP: injection discomfort is minimal and manageable

Among participants who mentioned discomfort or pain associated with receiving injections, the discomfort was described as mild to moderate and limited in duration.

*A little bit of pain, but it's an injection. It's expected. I've never been a needle person… but it's not bad. You get the injection, and then maybe 30 seconds later, you feel it running through your body*. – Non‐adherent, MSM, Domestic

Participants also identified strategies to manage pain and discomfort before, during and after injections, including medication and relaxation prior to injection visits, and massages, warm baths, stretching, walking and limiting high‐intensity exercise in the days post‐injection.

*Moving and walking around, and letting the muscle work and then relax, and work and then relax, is extremely helpful, with the soreness. It's when you've been stationary for too long, that's what the pain kicks in*. – Adherent, MSM, Domestic

*In the beginning, it was really tough because it was a pain that I was not used to. I received medication so that this pain could be eased, so my body got used to it… and nowadays I cope with it easily*. – Adherent, MSM, International

Another factor that decreased distress surrounding PrEP injections was a sense of personal autonomy and communication with study staff, particularly staff administering the injection. Having control over certain factors—for example, whether or not the nurse provided a verbal countdown before the injection itself—helped to reduce overall distress and anxiety.

*Usually there's always an option. “Do you want the one, two, three, or just the poke?” And I'm like, “Just give me the poke.” That's actually really important to know that they're listening*. – Adherent, MSM, Domestic

#### 3.2.5 Experiences with and perceptions of injectable PrEP: concerns and misperceptions about injection efficacy and safety were present

Some concerns were shared around the safety and efficacy of injectable PrEP. For example, one individual expressed a belief that injections would “wear off” prior to the next scheduled visit—and that, as a result, more frequent injections might be needed to maintain protection against HIV.

*It could wear off. If you come into contact with somebody that got [HIV], but the shot's supposed to fight it off, and it's been two months since you got a shot, the shot gonna be weak as hell. It ain't gonna do nothin’*. – Non‐adherent, MSM, Domestic

Other reported misconceptions included worrying that injectable PrEP might not work as well for individuals with a greater number of sexual partners compared to those with fewer partners, and for individuals who are more physically active.

*You can say, “Oh, it's potent” and “Oh, it'll last,” but it really depends. If you're a lazy person that gets a shot and sits home all day, it might stay in your system longer because you're not active… But if you somebody that's active, up every day, movin’ around, workin’… it's gonna wear off quicker because your body is steadily burnin’ energy*. – Non‐adherent, MSM, Domestic

Additionally, participants’ safety‐related concerns included the effects of injecting a “foreign” substance into their bodies, particularly one that would remain in their system for an extended period of time.

*Just the fact it's substance that I'm injecting into my body. The more foreign things you put into your body, that slows down the process of things. The fact that the liquid is yellow*,^1^ *I think about that*. – Non‐adherent, MSM, Domestic

*I'm generally a healthy person, so I never wanna put myself in a way that would compromise my health. When it comes to a shot, that lasts longer in your body, and once it's in there, it's just kinda like, girl, it's in there. But now, it's fine*. – Adherent, MSM, Domestic

#### 3.2.6 Facilitators of adherence to injectable PrEP: desire to preserve one's health functioned as a motivator for adherence to injectable PrEP

Participants across adherence categories reported that facilitators of their injection visit adherence centred around the desire to prevent HIV acquisition. Utilizing medication for prevention was viewed more favourably than using medication for HIV treatment:

*I don't wanna have [HIV]. There's two choices you have. You either try to prevent it or you can be taking a pill for it*. – Adherent, MSM, Domestic

*I was taking it to prevent [HIV], but I put myself in the place of a person that took it to keep healthy… and then I started wonder, “Man, imagine if I had HIV, having to take this medicine, not to have a healthy life, [but] to survive indeed” – and that moved me a bit*. – Adherent, MSM, International

Participants emphasized that, within the context of a double‐blind study, it was important to attend all injection visits to maximize the likelihood of protection from HIV.

*Being in a double blind study, I don't know if the pill is the pill. I don't know if the injection is the injection. That motivates me to come ‘cause I need to make sure that I'm still being healthy and safe across all spectrums*. – Adherent, MSM, Domestic

Several participants noted a responsibility to ensure the integrity of study findings, and that they were motivated to do so—both through maintaining adherence to the oral product throughout the study, as well as through their commitment to attending injection visits.

*It's important, even though… I'm with someone [and] I don't think I need the protection, but I still am part of the study, so I want to have respect for being part of the study and still be on top of everything*. – Non‐adherent, MSM, Domestic

#### 3.2.7 Facilitators of adherence to injectable PrEP: clinic staff and environment play a key role in facilitating attendance at injection visits and supporting adherence

Clinic‐related factors were critical to injection visit attendance—and, therefore, injectable PrEP adherence. These included the positive, affirming environment at research sites, relationships with and interpersonal qualities of study staff, and the open communication style and availability of staff to answer questions.

*If I had a negative experience here, it would make me not wanna come to my study more, but the fact that I had a really overall fantastic experience makes me more motivated to come here*. – Adherent, MSM, Domestic

Participants also reported that study staff across research sites went out of their way to be flexible with visit scheduling and respect participants’ competing demands—and that these accommodations facilitate their ability to attend injection visits. Participants highlighted that staff were exceptionally understanding of work‐related conflicts and frequently changing schedules, last‐minute rescheduling and travel.

*When I first started the study, I didn't travel as much for work as I do now. I thought that I wouldn't be able to do the study because of how much I'm on the road, but they've been able to be flexible and work around my schedule*. – Non‐adherent, MSM, Domestic

Participants also reported that both personal reminders, and clinic‐based reminders directly from study staff, were key facilitators of their injection visit adherence.

*They've been helpful, and they've been reminding me. I get text messages to remind me that my appointment is coming because, if not, I'll never make it*. – Early discontinuer, TGW, International

#### 3.2.8 Facilitators of adherence to injectable PrEP: benefit from social support around study participation

Finally, receiving support from other people—including reminders to attend injection visits, help with transportation, and demonstrating overall interest in their study participation—additionally facilitated participants’ adherence to injection visits.

*All my friends… Not that they help me directly, like a mother or father, but… every time they remember that I am participating, it is like, “Ah, how are you doing, when is the next appointment?”, etc*. – Early discontinuer, TGW, International

#### 3.2.9 Barriers to adherence to injectable PrEP: participants had to overcome structural barriers to attend study visits

Many barriers centred around structural and logistical factors related to transportation, including the travel distance to sites, inconvenience of clinic locations and related financial challenges (e.g. parking and gas costs). This was the case across different adherence categories.

*The one issue I can think of is more just logistical… it can take an hour plus to get down to [study site] by public transit. And I'm sure [study site] is much more convenient for some folks, but for me, it's not*. – Non‐adherent, MSM, Domestic

#### 3.2.10 Barriers to adherence to injectable PrEP: unpredictable work schedules and time away from employment were competing demands related to attending study visits

Participants across adherence categories frequently discussed challenges related to balancing injection visits with work commitments. Given busy and variable schedules, the ability to reschedule study visits was useful for injection visit adherence; however, in some cases, competing demands still required creative solutions.

*It was super easy to schedule when I was unemployed, but halfway through the study, I found regular employment and that was a little bit more difficult, but I always make it work because this is a necessity to me*. – Adherent, MSM, Domestic

For some, the extent to which work schedules represented a barrier to attending injection visits depended primarily on the flexibility of their employers.

*One time I was working… and I double‐booked this on top of that. And so I had to call off there and come here and they ended up putting me on suspension for two days and so I couldn't go to work for two days*. – Adherent, MSM, Domestic

The length of injection visits was also challenging for some participants in terms of managing work obligations alongside their desire to remain active in the study.

*Can we hurry this up? I don't wanna be here over an hour just to get a shot… I gotta go to work*. – Non‐adherent, MSM, Domestic

Finally, participants also discussed ways in which they balanced the competing demands of everyday life, including personal matters outside of work, with study participation. One participant described the complex reasons for stepping back from the study as follows:

*Recently, it was more complicated due to personal issues more than any other thing. I stayed away this time, three months… it is always a hurry to look after work, money and eating… that [the study] ended up not being prioritized… Not that I was not caring, but if I would choose between assuring my life and my well‐being or coming to the… appointment, I gave priority to this over coming here*. – Early discontinuer, TGW, International

## 4 DISCUSSION

Experiences in this injectable PrEP trial were generally positive, and study participation was viewed as a way to access a novel, free, potentially effective HIV prevention tool while also contributing to research. Participants reported some initial anxiety and misconceptions about injectable PrEP, as well as logistical and structural barriers to injection visit attendance; however, many concerns abated with time. Adherence was facilitated primarily by positive experiences with study staff and supportive clinic environments. Participants viewed injectable PrEP as a more convenient and less burdensome treatment option than daily oral PrEP—a finding consistent with recent discrete choice experiment studies examining PrEP preferences, which generally suggest a preference for long‐acting PrEP over daily formulations [13, 14, 15, 16, 17, 18].

Motivations to enrol in the parent trial and to adhere to injectable PrEP were similar. Participants described a desire to enhance and preserve their overall health, increase engagement in care and increase protection against HIV as important factors for both study participation and remaining adherent over time. These findings suggest that participants viewed injectable PrEP as an acceptable HIV prevention method and viewed the associated contact with healthcare professionals as a general health‐enhancing byproduct—an indirect or collateral benefit [19, 20]—of utilizing injectable PrEP within the clinical trial context, where care was viewed as compassionate, non‐judgemental and flexible. Participants also discussed additional reasons for participation, including the ability to access PrEP at no cost, which was especially important for those experiencing unemployment and/or other financial stressors.

Findings suggested some negative physical and psychological experiences during injectable PrEP initiation, which dissipated with time and coping strategies, including relaxation techniques, stretching and limiting strenuous exercise post‐injection. Some participants discussed initial injection‐related anxiety, fear of needles and worry around an investigational product, which decreased as the study progressed. This finding highlights the particular importance of retention at the outset of injectable PrEP care, when both injection‐related anxiety and the risk of discontinuation may be greatest [21]. It is also notable that much of the existing literature surrounding injectable PrEP comes from hypothetical users, and not individuals with real‐world experience [22, 23, 24]; indeed, many concerns about injections (e.g. persistent pain at the injection site) did not emerge as overly salient in this sub‐study. Relatedly, participants reported that having control over some injection‐related factors and the ability to communicate preferences to clinic staff (e.g. verbal countdown before injection) were important for reducing anxiety. One individual described early injections as triggering of past trauma (i.e. childhood sexual abuse). Given the disproportionate burden in the rates of violence, victimization and post‐traumatic stress disorder [25, 26, 27, 28, 29] borne by MSM and TGW, compared to cisgender and heterosexual individuals, this finding highlights the need for providers administering injectable PrEP to be trained in trauma‐informed care as well as management of injection‐related anxiety.

Some participants endorsed concerns about the efficacy and safety of injectable PrEP, as is appropriate for any new PrEP product and particularly within the trial context, where efficacy and safety must be established before more nuanced questions and concerns can be investigated. These concerns highlight opportunities for user‐ or client‐specific education around what is or is not known about basic product efficacy, forgiveness of missed doses, robustness to levels of sexual activity and/or incident ulcerative sexually transmitted infections (STIs), and whether physical activity influences either efficacy or safety. These concerns further underscore the importance of the provider−patient relationship in establishing a non‐judgemental environment with open communication, in which clients feel comfortable asking questions and providers are available to answer questions and provide education about injectable PrEP.

Clinic‐related factors—including the interpersonal qualities of study staff and the overall environment of study sites—were identified as key facilitators of injection visit adherence. Study staff were perceived to be compassionate, encouraging and non‐judgemental, and clinic environments were open and affirming spaces for sexual and gender minorities. In particular, participants reported that study staff members’ open communication styles, and availability to answer questions about PrEP and general health, played an important role in their adherence to injection visits—and often contrasted sharply with prior routine clinical care outside the research context. This is consistent with the Healthcare Provider Compassion Model [30], which highlights the combination of such interpersonal factors in providing effective healthcare to patients, and is also consistent with the Life‐Steps approach to adherence counselling [31]. Taken together, these findings suggest the importance of selecting for and training injectable PrEP providers in affirming, non‐judgemental and compassionate care, and using open communication styles to support patients’ adherence.

Some participants also reported utilizing social support to facilitate their adherence to injection visits. These findings extend the literature on the role of social support in medication adherence [32] and in PrEP use among MSM and TGW [33], highlighting the important role of friends and partners, rather than family members, and the specific role of instrumental social support.

Lastly, although participants reported mostly positive experiences, several barriers to injectable PrEP adherence were also identified, including difficulty managing work, personal and healthcare schedules, as well as accessing certain clinic locations. For some, changing and/or demanding work schedules were identified as a barrier to adherence, requiring participants to weigh attending injection visits with missing work or requesting time off. In addition to flexible scheduling, expanding clinic hours and offering injection appointments outside of traditional work hours and in non‐medical settings represent structural changes that would promote adherence to injectable PrEP. Participants also identified transportation to, and locations of, clinics as another barrier; clinic sites, which were frequently located at major medical centres, were often inconvenient and expensive to travel to (i.e. via public transit, taxis or gas and parking). This finding is consistent with extant literature from resource‐limited settings which has identified distance to a clinic as a major structural barrier to adherence [34, 35].

### 4.1 Limitations

Enrolment for this qualitative sub‐study was interrupted by the onset of the COVID‐19 pandemic, and then ultimately stopped prior to achieving the target sample size, due to the early DSMB discontinuation of the randomized blinded phase of the parent trial. In addition, although participants were purposively sampled across adherence categories in order to maximize potential variation in study experiences, most participants included in this sub‐sample (i.e. those from the three sites that conducted interviews prior to unblinding) belonged to the “adherent” (vs. “non‐adherent” or “early discontinuer”) category. In addition, most were participants from the United States, as only one international site (Rio de Janeiro, Brazil) had conducted any pre‐unblinding interviews (*n* = 4); the other two international sites (Cape Town, South Africa and Bangkok, Thailand) had not. Importantly, data from TGW participants are also limited in this sub‐sample (*n* = 2 TGW, and *n* = 2 gender queer or variant or non‐conforming). As such, overall, we were not able to achieve representative numbers from each adherence group, study site or TGW participants, reducing our ability to comment more fully on the experiences of those with varied levels of adherence, from other geographic locations, and with diverse gender identities. Moreover, particularly in light of the role that study staff, clinic environments and study‐related social support played in facilitating adherence for our participants, there may also be key differences in the barriers to and facilitators of injectable PrEP adherence outside the clinical trial context, which are not captured in these analyses.

## 5 CONCLUSIONS

These data on participant experiences in HPTN 083 using injectable and oral PrEP are the first of their kind, and provide insight for both future trials and implementation of injectable PrEP. Successful implementation may involve challenging paradigms of medical care. Infrastructure to help individuals negotiate financial constraints (inclusive of drug cost as well as the cost of getting to injection visits) and flexibility of scheduling will be critical to injectable PrEP implementation and ensuring continued access when biomedical HIV prevention is needed or desired. Settings that offer whole‐person care and general health screenings, and that have infrastructure to offer scheduling consistent with the reality of patients’ lives, is particularly important. Because many individuals at the highest risk for HIV are from stigmatized and minority populations, having staff who are open, affirming and willing to build relationships over time will be essential for the successful delivery of HIV prevention products. Lastly, providers should be aware of and inquire about injection‐related anxiety, including how injections may trigger traumatic memories, and should correct any misconceptions about how injectable PrEP (or the mechanisms of action of any HIV prevention product) works.

## COMPETING INTERESTS

JS received support from the National Institutes of Health for his work on this study. He is also a member of Merck KGaA's Ethics Advisory Panel and Stem Cell Research Oversight Committee; a member of IQVIA's Ethics Advisory Panel; a member of Aspen Neurosciences Clinical Advisory Panel; a member of a Merck Data Monitoring Committee; and a consultant to Biogen. None of these latter activities are related to the material discussed in this manuscript. CFK has received research grants to her institution from Gilead Sciences, ViiV Healthcare, Moderna, Novavax and Humanigen. RJL serves on a Scientific Advisory Board for Merck. ARR is an employee and shareholder of ViiV Healthcare. NP's institution received research funding from Gilead Sciences, consultant fees from Merck and advisory board fees from ViiV Healthcare (no direct payment to NP). All other authors declare that they have no competing interests.

## AUTHORS’ CONTRIBUTIONS

CP, SAS, BG and RJL conceptualized the study and designed the interview questions. WR, CFK, TO and LEC coordinated and performed data collection. CP, GRG and JSL conducted the qualitative analyses. CP and GRG drafted the manuscript. All authors critically revised the manuscript and approved its submission.

## FUNDING

Research reported in this publication was supported by the National Institute of Allergy and Infectious Diseases (NIAID), the National Institute of Mental Health (NIMH), the National Institute on Drug Abuse (NIDA), and the Eunice Kennedy Shriver Institute for Child Health and Human Development, under award numbers UM1AI068619 (HIV Prevention Trials Network [HPTN] Leadership and Operations Center), UM1AI068617 (HPTN Statistical and Data Management Center) and UM1AI068613 (HPTN Laboratory Center). ViiV Healthcare and Gilead Sciences donated trial medications and matching placebos, and ViiV Healthcare provided additional funding.

## DISCLAIMER

The content is solely the responsibility of the authors and does not necessarily represent the official views of the National Institutes of Health (NIH).

## Data Availability

The data that support the findings of this study are available from the corresponding author upon reasonable request.

## Footnotes

1 Some research sites used a yellow overlay on the syringe to mask any perceived differences between the active and placebo injections in the syringe.
